# Gene Annotation in High Schools: Successful Student Pipeline and Teacher Professional Development in Bioscience Using GENI-ACT

**DOI:** 10.3389/fmicb.2020.578747

**Published:** 2021-01-15

**Authors:** Stephen T. Koury, Shannon Carlin-Menter, Rama Dey-Rao, Kimberle Kelly

**Affiliations:** ^1^Department of Biotechnical and Clinical Laboratory Sciences, State University of New York at Buffalo, Buffalo, NY, United States; ^2^Department of Family Medicine, State University of New York at Buffalo, Buffalo, NY, United States; ^3^Oak Ridge Associated Universities, Oak Ridge, TN, United States

**Keywords:** professional development, STEM education and careers, curriculum development (education), high school (9–12), bioinformatics, gene annotation

## Abstract

Knowledge of genomics is an essential component of science for high school student health literacy. However, few high school teachers have received genomics training or any guidance on how to teach the subject to their students. This project explored the impact of a genomics and bioinformatics research pipeline for high school teachers and students using an introduction to genome annotation research as the catalyst. The Western New York-based project had three major components: (1) a summer teacher professional development workshop to introduce genome annotation research, (2) teacher-guided student genome annotation group projects during the school year, (3) with an end of the academic year capstone symposium to showcase student work in a poster session. Both teachers and students performed manual gene annotations using an online annotation toolkit known as Genomics Education National Initiative-Annotation Collaboration Toolkit (GENI-ACT), originally developed for use in a college undergraduate teaching environment. During the school year, students were asked to evaluate the data they had collected, formulate a hypothesis about the correctness of the computer pipeline annotation, and present the data to support their conclusions in poster form at the symposium. Evaluation of the project documented increased content knowledge in basic genomics and bioinformatics as well as increased confidence in using tools and the scientific process using GENI-ACT, thus demonstrating that high school students are capable of using the same tools as scientists to conduct a real-world research task.

## Introduction

With the continuing expansion of genomic databases, discovery of rare disease-causing genetic variations and reports of drug efficacy-genotype associations, genomics has ever-increasing relevance to everyday life. It is important that the education of everyone, from doctors to patients, include genomics and bioinformatics for the continued successful integration of genomics into healthcare (Green et al., 2011). At the same time, career opportunities for students trained in genomics are growing and the recruitment and retention of talent in genomics is important for United States economic growth (Grand View Research, 2019). This growth is due to technical advances, with DNA sequence data being generated at a much faster rate, which has created a gap between the actual generation of data and its analysis (Li et al., 2016).

While a thorough knowledge of genomics is an essential component of science and health literacy required for students to become informed citizens, consumer and professionals, educational resources and curricula fail to address this need, as few high school teachers have received genomics training or any guidance on how to teach the subject to their students (Wray, 2017). Even fewer resources are available to high school teachers to address the newer, nuanced understanding of genome structure and function and emerging genomic technologies, such as genome sequencing (National Human Genome Research Institute, 2018). The Next Generation Science Standards (NGSS) promote a three-dimensional learning approach focused on core ideas intertwined with science and engineering practices and cross-cutting concepts such as “structure and function” (Next Generation Science Standards, 2019) and the AP Biology curriculum has been redesigned to incorporate inquiry-driven scientific practices in the core (Anon, 2019). These changes in standards provide an opportunity to embed more genomics into the high school classroom, involving students in applications of genomics in real-world problem-solving settings. Incorporating inquiry-based genetic sequencing science projects into the high school curriculum is a way to narrow this knowledge gap and to educate, inspire and encourage the development of technical research skills that are needed within healthcare and personalized genomics (Ditty et al., 2010; Moitra, 2017).

### Project Background

Beginning in 2013 and funded by a 3-years NSF Innovative Technology Experiences for Students and Teachers (ITEST) Grant, we developed the Western New York Genetics in Research Partnership (WNYGRP). The partnership was comprised of the University at Buffalo, including the departments of Biotechnical and Clinical Laboratory Sciences and Family Medicine; the NYS Center of Excellence in Bioinformatics and Life Sciences (CBLS); the New York State Area Health Education Center System (NYSAHEC), including Erie-Niagara (EN AHEC) and Western New York Rural (R-AHEC); Oak Ridge Associated Universities (ORAU); UB faculty with expertise in genome annotation; and a NYS STEM Master High School Teacher. The project introduced high school teachers and students to genomics and bioinformatics through the use of freely available, hands-on, state-of-the-art bioinformatics tools.

This ITEST research project developed partnerships with disadvantaged high schools across a 14-county region in Western New York, forming a pipeline for teacher and student recruitment. The details of the development of the partnership will be presented elsewhere. Grades 9–12 biology teachers were trained on the use of the Genomics Education National Initiative-Annotation Collaboration Toolkit (GENI-ACT)^1^. This innovative technology experience increased high school students’ and teachers’ knowledge of bioinformatics and allowed teachers to gain experience with bioinformatics software tools for classroom use through real-world research experiences.

## Program Components

The ITEST project had three major components outlined below, consisting of a summer teacher professional development (PD) workshop, teacher-guided student genome annotation projects during the school year, and a capstone symposium at the end of the school year. High school Biology teachers recruited from the targeted schools signed-up for the summer workshop for a variety of reasons, including learning something new, using the training hours to count toward their mandatory staff development, the stipend they received for their involvement, and/or the ability to offer their students something new to add to their portfolios or highlight during college interviews. One teacher commented, “The idea of exposing students to real science was very enticing to me and I feel like the idea of being a scientist and being able to handle Big Data is a skill that we need to start teaching our students.” Overall, we recruited 74 Biology teachers over the 3 years to take part in the summer professional development training.

### Summer PD Workshop

During the 5-day Summer Workshop, teachers were trained using nine modules customized by project faculty that were based on those in GENI-ACT (9, Table 1). After the training, the teachers worked with their students on the same modules during the school year. GENI-ACT and the online bioinformatics tools utilized during the training were free, so only computer and internet access were needed to take part in the project. First, we presented teachers with background knowledge that provided them with an understanding of genomics, DNA structure, and transcription/translation relevant to gene annotation. Teachers were then instructed on how to log into GENI-ACT and navigate the website.

**TABLE 1:** The modules used in GENI-ACT.

Modules	Activities	Questions investigated
Basic information	DNA Coordinates and Sequence, Protein Sequence	What is the sequence of the gene and protein? Where is it located in the genome?
Sequence-based similarity	Blast (Altschul et al., 1990), COG (Tatusov et al., 1997), T-Coffee (Di Tommaso et al., 2011), WebLogo (Crooks et al., 2004)	How similar is the sequence of the protein under investigation to other proteins in GenBank?
Structure-based similarity	TIGRFAM (Haft et al., 2001), Pfam (El-Gebali et al., 2019), PDB (Berman et al., 2000)	What functional domains are present in the protein under investigation?
Cellular localization	Gram Stain, TMHMM (Krogh et al., 2001), SignalP (Almagro Armenteros et al., 2019), PSORTb (Yu et al., 2010), Phobius (Käll et al., 2007)	Is the protein under investigation located in the cytoplasm, secreted, in the periplasm, or embedded in the cell membrane or cell wall?
Enzymatic function	KEGG (Kanehisa and Goto, 2000), MetaCyc (Karp et al., 2002), E.C. Number (Expasy, 2020)	In what process or structure is the protein under investigation involved?
Duplication and degradation	Paralog, Pseudogene	Are there other forms of the protein under investigation in the same genome? Is it functional?
Horizontal gene transfer	Phylogenetic Tree, Gene Neighborhood, GC Content	Has the protein under investigation co-evolved with the rest of the genome or has it been obtained in a different way?
RNA family	Rfam (Kalvari et al., 2018)	Does the gene under investigation encode a functional RNA?
Final annotation	Evaluate data from all modules	Has the gene been correctly called by the pipeline annotation?

*GENI-ACT was undergoing a transition at the time the project was initiated, resulting in creation of customized notebooks and instructions for this project (NSF, 2020).*

Faculty instructors assigned the teachers a set of demonstration genes to annotate that illustrated positive and negative results obtained from the tools in the modules. Teachers were shown how to use each tool and interpret results using such parameters as scores and *e*-values and then allowed to apply it on their own during the week of training. The relative strengths and drawbacks of results obtained from different databases were stressed to inform the development of hypotheses about genes under investigation.

A manual with background information and complete step-by-step instructions for completing all modules was developed during the project is freely available on our website (NSF, 2020). The gene annotation work was interspersed with talks from project faculty on personalized genomics and program evaluation. Teachers completed pre and post-workshop surveys to evaluate gains in content knowledge about bioinformatics related to genome annotation and their comfort level with teaching bioinformatics concepts.

### Academic Year Annotation Projects

As the teachers returned to school in September, they recruited student participants and trained them using the nine GENI-ACT modules. All interested students were offered career counseling and exposure to genomics activities to encourage the recruitment of student participants. Activity 1, College and Career Exploration, was facilitated by AHEC coordinators from the school’s local center, R-AHEC or EN-AHEC, and provided students with STEM college and career guidance. Activity 2, also facilitated by AHEC, explored bioinformatics and genomic careers in more detail. Activity 3, facilitated by University of Buffalo faculty, provided students with an introduction to genome annotation. A total of 1,948 high school students attended at least one of the three activities over the 3 years of the program.

To evaluate the effectiveness of the program, informed consent was obtained from all participating students, and pre and post surveys assessed gains of student knowledge and changes related to their attitudes about careers in STEM. An experimental design was used, which randomized the 667 students recruited by the teachers into two groups: 343 were randomized into the intervention group (received GENI-ACT training) and the other 324 into the comparison group (no GENI-ACT training). Comparison group activities included various topics, which included researching bioethics or doing background research on genes identified by the annotators and/or the organism under study. Each student group in the intervention (GENI-ACT trained) was assigned a unique gene from the bacterium *Kytococcus sedentarius.* The students worked on this gene in the modules, along with a demonstration gene that teachers could use in a “show one, do one” model of teaching. Most teachers worked with their students through an after school club, as teachers were compensated for their time outside the classroom. Since a randomized design was utilized, the control and intervention students’ work were separated and easier to control outside of the regular classroom in an after school program. On average, teachers met with their intervention students once a week from January through April of the school year. Each teacher worked with a group averaging about seven students, assisting their work on the modules and recording data in their online notebooks. The students enjoyed the GENI-ACT modules. As one student explained “the modules themselves along with the paper manual really made the program easy to follow, which was great for first time students.” Students also appreciated that each of the genes they were assigned were different and that the modules allowed them find something unique about their particular gene. One student commented that the aspect of the uniquely assigned genes helped to fuel their love of research.

**FIGURE 1:**
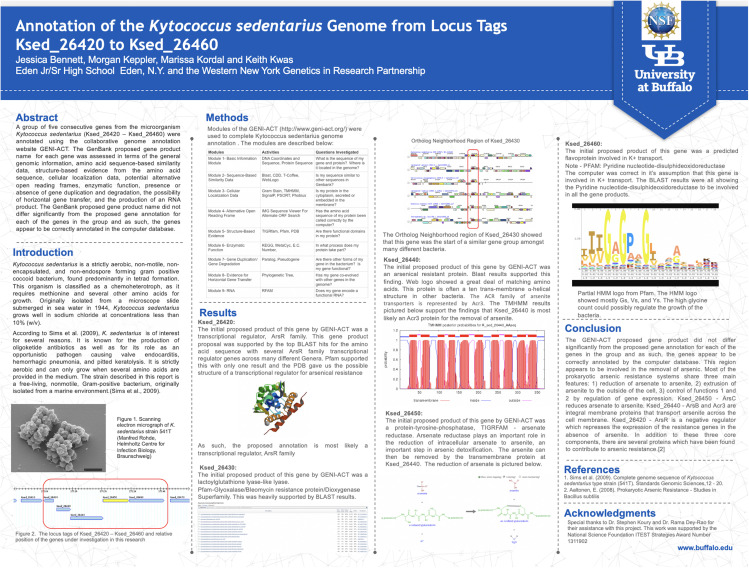
Example of a capstone event poster. Two to three students typically pooled data to prepared a poster in most instances and took turns presenting the poster at the capstone. High resolution versions of all posters presented at capstone events are available on the Student Research page of our project website (NSF, 2020).

Refresher trainings were offered to teachers on three different Saturdays during the school year. The third refresher training, offered in April, dealt with preparing the teachers for their students’ research poster preparation and presentation at the project culminating Capstone Symposium held in May. Using a poster template that could be populated with data generated by their students, teachers submitted the completed posters to program faculty approximately 1 week before the capstone, and faculty edited them for formatting only (Figure 1). The content was left as submitted (unless a glaring error was noted) to ensure that the posters represented student work and data interpretation. All posters were printed with dimensions of 4 × 3 feet and displayed at the capstone symposium.

### Capstone Symposium

In all, four student capstones were hosted. A total of 136 posters were prepared and presented during capstone symposia from 2014 to 2017 and are viewable on our online website (NSF, 2020). Annual Capstone Symposia took place at the end of each project year at the University at Buffalo, and, on two different occasions in academic institutions outside of the immediate Buffalo area, with participant numbers increasing each successive year. The capstone provided each student participant with the experience of attending a scientific meeting to present their data and to network with other teacher/student participants and program faculty. The capstone poster session was broken into two sections, allowing students to visit and interact with students from other schools.

A luncheon also allowed for informal interaction among students, followed by a series of speakers highlighting current topics in bioinformatics and genomics. The capstones concluded with a ceremony recognizing each student and teacher participant with a certificate of participation. Teachers were encouraged to take their posters back to their school and display them in the hallway or classroom. One teacher commented that their students “are very proud of those posters hanging up there in the hallway.” Another teacher noted that the capstone is “a nice program for the high school students to see what’s going on at the college level and the poster event is something unique, and something we don’t usually do at the high school level.”

### Program Outcomes

Teacher Content Knowledge was measured before and after the workshop. Teachers were asked to complete two sets of 10 True/False questions to assess their knowledge of bioinformatics and genome annotations at the start and end of the summer training workshop. The ten questions included in Set 1 were developed by the Microbial Genome Annotation Network (MGAN) to assess learning in students who used GENI-ACT within their courses. Set 2 includes 10 supplemental items developed by Faculty to help assess learning specific to the program. Mixed ANOVAs produced a significant increase in content knowledge scores from the pre workshop survey to the post workshop survey [*F*(1,31) = 37.86, *p* < 0.001, η^2^*p* = 0.55], confirming that teachers increased their content knowledge of bioinformatics and gene annotation by the end of the workshop, as predicted. The content knowledge questions, scoring, and example teacher responses are available in the educational resources section of our project website (NSF, 2020a).

Teaching Behaviors around bioinformatics and gene annotation were also expected to increase as a result of training. As a way of gauging their comfort with teaching the material, teachers were asked to rate their confidence in teaching GENI-ACT content topics. Specifically, teachers rated 28 topics on a percentage scale, from 10 to 100% in 10-percentage point increments. Their pre and post workshop ratings were compared using paired *t*-tests. In the case of every single topic, there was a significant increase by the end of the workshop. The mean increase in confidence from pre workshop to post workshop across all 28 content topics was 56%. The workshops clearly prepared teachers to use the GENI-ACT content and software tools with their students. However, not all teachers went on to work with students during the following academic year, with reasons including perceived difficulty of the project activities, difficulty implementing the study using the control group model or that they personally did not want to participate in the project.

Student content knowledge was projected to increase by the end of program in the intervention group, or those students receiving training on the GENI-ACT modules. Students completed the same content knowledge assessment as the teachers, measured twice as part of pre and post student surveys. Students were asked to complete two sets of 10 True/False questions to assess their knowledge of bioinformatics and genome annotations. In independent *t*-tests, Intervention students significantly increased their content knowledge of bioinformatics and gene annotation by the end of the project, while comparison students did not, on both Set 1, *t*(173) = 3.19, *p* = 0.002 and Set 2, *t*(173) = 8.40, *p* < 0.001. Moreover, the scores in the Treatment group increased by well over 50%, especially in Knowledge Set 2.

### Participant Perspectives

Impact of the project could be seen in student participants when it came to college applications, choosing a major and college interviews. One student said that “After participating in the ITEST program I knew that I wanted to become a chemical engineer. Furthermore, I knew that I wanted to attend the University at Buffalo because of how research-oriented the university is. Lastly, I knew that I wanted to attempt to pursue applications of chemical engineering in medicine and specifically the genomic medicine field. Over the next 4 years and beyond, I plan to pursue a career in this field.” Another student, who was accepted into RIT after participating in this program, was able to petition to be allowed into a Bioinformatics course that was only available for seniors as an elective. He was able to take the course as a Sophomore because he was able to prove through his Capstone poster that he had all the background knowledge to take the course.

Other teacher and student perspectives on performing gene annotations as a part of this project are available in an NSF STEM For All Video Showcase presentation (Videohall, 2016).

## Discussion

The results of this project informed different approaches to gene annotation with high school students and teachers that were utilized in another recently completed NIH Science Education Partnership Award (manuscript in preparation). The valuable partnership relationships developed have continued to expand since completion of the ITEST project described here and continue for the foreseeable future through another recently funded project. This project demonstrated that grade 9–12 students could grasp gene annotation and bioinformatics tools and use them appropriately.

The major limitation of this project for teachers was the use of the control group design. With this design, teachers could not include the gene annotation activities within their regular classes due to the need of having some students in a control group. This restricted most teachers to working with students before or after regular school hours, resulting in competition with other after-school student activities (sports/clubs). Another limitation of the control group design was the amount of time needed to recruit and randomize students before they could begin working with students on their annotations. As such, most teachers could not to begin work with their students until well after winter break and were only able to work through the first four modules before the end of the school year.

Sustained use of the bioinformatics tools by teacher participants after project completion is being explored and will be reported in more detail elsewhere. While complete gene annotation is not a common theme, teachers have been able to pick and choose tools from modules to integrate into their curriculum with relative ease. Some teachers have continued to pursue complete gene annotations and have their students present at the annual capstone event tied to another project, as they feel the poster presentation is a great experience for their students. One past participating teacher has integrated all nine GENI-ACT modules into his Honors Biology class by putting together PowerPoint presentations based on the Modules and meeting with the students every day in a lab situation. Future research might aim to determine the effect of taking part in gene annotation on academic performance related to biology and genetics. A study performed at the community college level demonstrated that students taking part in gene annotation in a cell biology lab exhibited clear gains in understanding of topics related to molecular biology in a lecture course (Beagley, 2013), suggesting similar gains could be expected in the high school classroom as well. Additional research is needed to identify topics most appropriate for, and learned most optimally by, high school students. For example, which aspects of bioinformatics-based research would most easily be integrated into high school biology curricula guided by NGSS? NGSS-friendly curricula will make it easier for teachers to introduce more students to bioinformatics. While bioinformatics software tools are complex and their use is challenging to teach, this study shows they can be successfully used by high school teachers with their students. Furthermore, utilizing the same bioinformatics tools used by scientists to conduct authentic research promotes student interest in science by seeing that they too can apply the scientific method to study real-world problems.

## Data Availability Statement

The raw data supporting the conclusions of this article will be made available by the authors, without undue reservation.

## Ethics Statement

The studies involving human participants were reviewed and approved by the University at Buffalo (IRB). Written informed consent to participate in this study was provided by the participants’ legal guardian/next of kin.

## Author Contributions

SK: training teachers, working with high school students, editing student posters, and writing the manuscript. RD-R: training teachers, working with high school students, and editing student posters. SC-M: program evaluation, writing the manuscript, and supervision of program manager. KK: program evaluation and writing the manuscript. All authors contributed to the article and approved the submitted version.

## Conflict of Interest

The authors declare that the research was conducted in the absence of any commercial or financial relationships that could be construed as a potential conflict of interest.

## Footnotes

1 https://geni-act.org

## References

[B1] Almagro Armenteros J. J. Tsirigos K. D. Sønderby C. K. Petersen T. N. Winther O. Brunak S. (2019). SignalP 5.0 improves signal peptide predictions using deep neural networks. *Nat. Biotechnol.* 37 420–423. 10.1038/s41587-019-0036-z 30778233

[B2] Altschul S. F. Gish W. Miller W. Myers E. W. Lipman D. J. (1990). Basic local alignment search tool. *J. Mol. Biol.* 215 403–410. 2231712 10.1016/S0022-2836(05)80360-2

[B3] Anon (2019). *AP Biology Investigative Labs: An Inquiry-Based Approach. 2012.* Available from: https://secure-media.collegeboard.org/digitalServices/pdf/ap/APBioTeacherLabManual2012_2ndPrt_lkd.pdf (accessed June 29, 2019).

[B4] Beagley C. T. (2013). Genome annotation in a community college cell biology lab. *Biochem. Mol. Biol. Educ.* 41 44–49. 10.1002/bmb.20669 23382125

[B5] Berman H. M. Westbrook J. Feng Z. Gilliland G. Bhat T. N. Weissig H. (2000). The protein data bank. *Nucleic Acids Res.* 28 235–242. 10592235 10.1093/nar/28.1.235 PMC102472

[B6] Crooks G. E. Hon G. Chandonia J. M. Brenner S. E. (2004). WebLogo: a sequence logo generator. *Genome Res.* 14 1188–1190. 10.1101/gr.849004 15173120 PMC419797

[B7] Di Tommaso P. Moretti S. Xenarios I. Orobitg M. Montanyola A. Chang J. M. (2011). T-Coffee: a web server for the multiple sequence alignment of protein and RNA sequences using structural information and homology extension. *Nucleic Acids Res.* 39 W13–W17. 10.1093/nar/gkr245 21558174 PMC3125728

[B8] Ditty J. L. Kvaal C. A. Goodner B. Freyermuth S. K. Bailey C. Britton R. A. (2010). Incorporating genomics and bioinformatics across the life sciences curriculum. *PLoS Biol.* 8:e1000448. 10.1371/journal.pbio.1000448 20711478 PMC2919421

[B9] El-Gebali S. Mistry J. Bateman A. Eddy S. R. Luciani A. Potter S. C. (2019). The Pfam protein families database in 2019. *Nucleic Acids Res.* 47 D427–D432. 10.1093/nar/gky995 30357350 PMC6324024

[B10] Expasy (2020). *Expasy Enzyme.* Available online at: https://enzyme.expasy.org (accessed June 30, 2020).

[B11] Grand View Research (2019). *Genomics Market Size, Share & Trends Analysis Report By Application And Technology (Pathway Analysis, Functional Genomics), By Deliverables (Instruments, Consumables, Services), By End Use, And Segment Forecasts, 2019 - 2025. 2019.* Available online at: https://www.grandviewresearch.com/industry-analysis/genomics-market (accessed June 25, 2020).

[B12] Green E. D. Guyer M. S. National Human Genome Research Institute (2011). Charting a course for genomic medicine from base pairs to bedside. *Nature* 470:204. 10.1038/nature09764 21307933

[B13] Haft D. H. Loftus B. J. Richardson D. L. Yang F. Eisen J. A. Paulsen I. T. (2001). TIGRFAMs: a protein family resource for the functional identification of proteins. *Nucleic Acids Res.* 29 41–43. 10.1093/nar/29.1.41 11125044 PMC29844

[B14] Käll L. Krogh A. Sonnhammer E. L. (2007). Advantages of combined transmembrane topology and signal peptide prediction–the Phobius web server. *Nucleic Acids Res.* 35 W429–W432. 10.1093/nar/gkm256 17483518 PMC1933244

[B15] Kalvari J. Argasinska J. Quinones-Olvera N. Nawrocki E. P. Rivas E. Eddy S. R. (2018). Rfam 13.0: shifting to a genome-centric resource for non-coding RNA families. *Nucleic Acids Res.* 37 420–423. 10.1093/nar/gkx1038 29112718 PMC5753348

[B16] Kanehisa M. Goto S. (2000). KEGG. Kyoto rncyclopedia of genes and genomes. *Nucleic Acids Res.* 28 27–30. 10592173 10.1093/nar/28.1.27 PMC102409

[B17] Karp P. D. Riley M. Paley S. M. Pellegrini-Toole A. (2002). The MetaCyc database. *Nucleic Acids Res*. 30 59–61. 10.1093/nar/30.1.59 11752254 PMC99148

[B18] Krogh A. Larsson B. von Heijne G. Sonnhammer E. L. L. (2001). Predicting transmembrane protein topology with a hidden Markov model: application to complete genomes. *J. Mol. Biol.* 305 567–580. 10.1006/jmbi.2000.4315 11152613

[B19] Li J. Batcha A. M. Grüning B. Mansmann U. R. (2016). An NGS workflow blueprint for DNA sequencing data and its application in individualized molecular oncology. *Cancer Inform.* 14(Suppl. 5), 87–107. 27081306 10.4137/CIN.S30793 PMC4827795

[B20] Moitra K. (2017). Releasing the “GENI”: integrating authentic microbial genomics research into the classroom through GENI-ACT. *FEMS Microbiol. Lett.* 364:fnx215. 10.1093/femsle/fnx215 29040493

[B21] National Human Genome Research Institute (2018). *Genomic Literacy, Education, And Engagement (Glee) Initiative 2017 Strategic Visioning Meeting: K-16 Working Group. 2017 2018.* Available online at: https://www.genome.gov/Pages/About/OD/ECIB/GLEE/GLEE_white_paper_K-16_WG.pdf (accessed June 25, 2018).

[B22] Next Generation Science Standards (2019). Available from: https://www.nextgenscience.org (accessed June 29, 2019).

[B23] NSF (2020). *Western New York Genetics in Research Partnership Educational Resources.* Available from: http://ubwp.buffalo.edu/wnygirp/educational-resources/ (accessed June 26, 2020).

[B24] Tatusov R. L. Koonin E. V. Lipman D. J. (1997). A genomic perspective on protein families. *Science* 278 631–637. 10.1126/science.278.5338.631 9381173

[B25] Videohall (2016). *Western New York Genetice in Research Partnership NSF STEM For All Video Showcase.* Avaialble at: https://stemforall2016.videohall.com/presentations/709 (accessed June 26, 2020).

[B26] Wray C. G. (2017). Introducing students to the genome: brave new world or the red Queen’s wonderland? *Am. Biol. Teach.* 79 253–253. 10.1525/abt.2017.79.4.253 33021500

[B27] Yu N. Y. Wagner J. R. Laird M. R. Melli G. Rey S. Lo R. (2010). PSORTb 3.0: improved protein subcellular localization prediction with refined localization subcategories and predictive capabilities for all prokaryotes. *Bioinformatics* 26 1608–1615. 10.1093/bioinformatics/btq249 20472543 PMC2887053

